# A new CMR protocol for non-destructive, high resolution, ex-vivo assessment of the area at risk simultaneous with infarction: validation with histopathology

**DOI:** 10.1186/1532-429X-14-S1-O7

**Published:** 2012-02-01

**Authors:** Lowie M Van Assche, Han W Kim, Christoph J Jensen, Ki-Young Kim, Michele Parker, Raymond J Kim

**Affiliations:** 1 Cardiology, Duke University, Durham, NC, USA

## Background

In the setting of acute myocardial infarction (AMI), the aim of reperfusion and pharmacologic therapies is to salvage areas of ischemic, but reversibly injured myocardium within the area-at-risk (AAR). The current histopathologic reference standard requires administration of microspheres, destructive sectioning of the heart, the use of tissue stains, and digital photography to delineate the AAR, infarction, and to calculate salvage. We evaluated a newly developed CMR protocol that potentially provides non-destructive, high-resolution, ex-vivo assessment of the AAR simultaneous with infarction.

## Methods

Four canines underwent 50-minute occlusion of the LAD coronary followed by reperfusion. Imaging was performed 5-days post-AMI. The main goal of the protocol was to create 3 distinct myocardial gadolinium concentrations delineating viable AAR, infarcted AAR and remote myocardium. This was achieved by (1) injecting gadolinium (0.3-0.4mmol/kg) in-vivo to differentially accumulate in infarction, (2) providing a wait time to allow washout of gadolinium from viable myocardium, (3) injecting another dose of gadolinium prior to sacrifice using the same process as if administering microspheres for determining the AAR by pathology. For validation purposes, microspheres (2-8μm, ThermoScientific) were mixed with gadolinium in this step. Finally, (4) the heart was extracted and ex-vivo 3-dimensional-delayed-enhancement-CMR imaging was performed. The heart was sectioned into short-axis slices and photographed under UV-light to delineate the AAR (absence of microspheres) and after staining with triphenyltetrazoliumchloride to delineate infarction. Histopathology and ex-vivo-CMR images were analyzed by 2 blinded observers.

## Results

**Figure 1:**
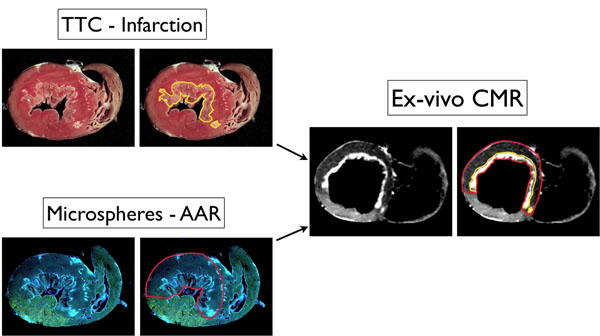
Example of sub-endocardial AMI with transmural risk region.

Figure 1 shows histopathology of a subendocardial-AMI with transmural AAR. On the matched delayed-enhancement-CMR image, 3 distinct image intensities are present: (1) myocardium with lowest signal matches location and shape of the viable AAR by microspheres, (2) region with highest signal matches infarction on pathology and (3) remote zone with intermediate signal.

On an animal basis, ex-vivo-CMR AAR was similar to that by microspheres (33%±7 vs. 35%±6 respectively, p=0.4). On a slice basis (n=27), there was a strong linear correlation between the ex-vivo-CMR and microsphere-defined AAR (r=0.98, slope=0.96±0.04, p<00001). Similarly, CMR infarct size matched that by triphenyltetrazoliumchloride (r=0.97, p<0.0001). Calculated CMR salvage was also highly correlated with that of histopathology (r=0.92, p<0.0001).

## Conclusions

We developed a new CMR protocol that provides high-resolution, ex-vivo images of the AAR simultaneous with infarction in the same 3-dimensional dataset. This can serve as an alternative to histopathology as a truth standard measurement of the AAR and salvage that is non-destructive, allows for multiplanar reconstruction and is automatically registered with the spatial map of infarction.

## Funding

Funded in part by 5R01HL064726-07.

